# *Enterocytozoon schreckii* n. sp. Infects the Enterocytes of Adult Chinook Salmon (*Oncorhynchus tshawytscha*) and May Be a Sentinel of Immunosenescence

**DOI:** 10.1128/msphere.00908-21

**Published:** 2022-01-05

**Authors:** Claire E. Couch, Michael L. Kent, Louis M. Weiss, Peter M. Takvorian, Stephanie Nervino, Leslie Cummins, Justin L. Sanders

**Affiliations:** a Department of Fisheries, Wildlife, and Conservation Sciences, Oregon State University, Corvallis, Oregon, USA; b Department of Microbiology, Oregon State University, Corvallis, Oregon, USA; c Departments of Pathology and Medicine, Albert Einstein College of Medicine, Bronx, New York, USA; d Department of Biological Sciences, Rutgers University, Newark, New Jersey, USA; e Department of Biomedical Sciences, Oregon State University, Corvallis, Oregon, USA; University at Buffalo

**Keywords:** Enterocytozoon, Enterospora, immunosuppression, microsporidia, salmon

## Abstract

A novel *Enterocytozoon* infection was identified in the intestines of sexually mature Chinook salmon. While microsporidian parasites are common across a diverse range of animal hosts, this novel species is remarkable because it demonstrates biological, pathological, and genetic similarity with Enterocytozoon bieneusi, the most common causative agent of microsporidiosis in AIDS patients. There are similarities in the immune and endocrine processes of sexually mature Pacific salmon and immunocompromised humans, suggesting possible common mechanisms of susceptibility in these two highly divergent host species. The discovery of Enterocytozoon schreckii n. sp. contributes to clarifying the phylogenetic relationships within family Enterocytozoonidae. The phylogenetic and morphological features of this species support the redescription of *Enterocytozoon* to include Enterospora as a junior synonym. Furthermore, the discovery of this novel parasite may have important implications for conservation, as it could be a sentinel of immune suppression, disease, and prespawning mortality in threatened populations of salmonids.

**IMPORTANCE** In this work, we describe a new microsporidian species that infects the enterocytes of Chinook salmon. This novel pathogen is closely related to Enterocytozoon bieneusi, an opportunistic pathogen commonly found in AIDS patients and other severely immunocompromised humans. The discovery of this novel pathogen is of interest because it has only been found in sexually mature Chinook salmon, which have compromised immune systems due to the stresses of migration and maturation and which share similar pathological features with immunocompromised and senescent humans. The discovery of this novel pathogen could lead to new insights regarding how microsporidiosis relates to immunosuppression across animal hosts.

## INTRODUCTION

Microsporidia are a widespread, diverse group of spore-forming intracellular parasites that can cause severe disease (1–3) but can also develop long-term relationships with their hosts (4), persisting as subclinical latent infections. During the early HIV/AIDS pandemic in the 1980s, it was recognized that microsporidiosis in immune-suppressed humans can cause significant morbidity and mortality presenting as chronic diarrhea and wasting syndrome (5, 6). Prior to modern anti-retroviral therapies, intestinal microsporidiosis caused by Enterocytozoon bieneusi infection of enterocytes was observed in about 30% of HIV/AIDS patients (confidence interval, 15 to 85%) (7). Infection has since been observed in the elderly as well as other individuals experiencing accelerated immune senescence (2). Transient infections of E. bieneusi have also been detected in immunocompetent individuals after travel (8) or after exposure to contaminated foods (9), but in immunocompetent hosts, symptoms rarely persist longer than a few weeks even when chronic, subclinical infection occurs (10).

E. bieneusi is the type species of the family Enterocytozoonidae, and since its discovery, a number of other species and genera belonging to this family have been described in aquatic and terrestrial hosts (2). However, little is known about the possible role of host immune senescence in the progression of Enterocytozoonidae or other microsporidian infections of nonhuman animals. Indeed, the phenomenon of immunosenescence is poorly understood in most animals (11, 12), despite its potential significance for host disease, fitness, and population dynamics. Current understanding of microsporidiosis in humans and laboratory model organisms (13) suggests that susceptibility to microsporidian infection could serve as an indicator of immune pathology and senescence across species.

Chinook salmon (Oncorhynchus tshawytscha) belong to one of the relatively few groups of semelparous vertebrates that predictably undergo rapid senescence and death following their first and only reproduction event (14). This species has a long, complex life cycle that includes multiple physical transformations to match environmental and life history demands. As with other species of Pacific salmon, Chinook salmon hatch and rear in freshwater streams and then undergo dramatic physiological and morphological changes that allow them to transition from freshwater to saltwater as they migrate to the ocean. After 1 to 7 years of feeding on rich marine resources, adults migrate upriver to their natal streams where they undergo rapid senescence and ultimately spawn and die. The period of rapid senescence prior to spawning is accompanied by increased susceptibility to parasites and pathogens (15, 16), likely driven at least in part by physiological shifts that mirror those associated with immune deficiency in humans, including reduction of available energy (17, 18), increase in baseline cortisol (19, 20), and alterations to immunity (21, 22). Pacific salmon (23) and other teleost fishes (24) undergo involution of the thymus as they reach sexual maturity, mimicking the thymic involution of HIV-infected children and adults and potentially altering T-cell composition (25).

Many populations of Chinook and other Pacific salmon are threatened by high rates of prespawning mortality (PSM), which occurs when fish successfully complete migration but prematurely senesce and die before spawning can occur. In the Willamette River basin of Oregon, PSM in spring Chinook can exceed 95% in certain areas, severely threatening genetic diversity and population persistence. The influence of immune senescence and disease on PSM in these fish is unclear; high burdens of common parasites are observed in fish that spawn successfully as well as those that undergo PSM. However, adults held in cool, pathogen-free water prior to spawning are less likely to undergo PSM (16), suggesting that disease and pathogen burden may interact with elevated temperature and other stressors to increase the likelihood of PSM. Because pathogen dynamics can be affected by flow regimes and other watershed management actions, characterizing the suite of parasites and pathogens infecting adult Chinook salmon is of great importance to informing adaptive management.

A number of parasites infect adult Chinook salmon, including bacteria (e.g., Aeromonas salmonicida, Renibacterium salmoninarum), myxosporea (e.g., Parvicapsula minibicornis, Ceratonova shasta, Myxobolus sp.), trematodes (e.g., Nanophyetus salmincola, Apophallus sp., Echinochasmus milvi), and copepods (e.g., Salmicola californiensis). During routine histological surveillance for these parasites, we identified a novel Enterocytozoon-like infection in the intestines of sexually mature fish. While microsporidian parasites are common across a diverse range of animal hosts, this novel species is remarkable because it demonstrates biological, pathological, and genetic similarity with E. bieneusi, the most common causative agent of microsporidiosis in AIDS patients. Similarities in the immune and endocrine processes of senescent Pacific salmon and immunocompromised humans suggest common mechanisms of susceptibility in these two highly divergent host species.

## RESULTS

### Imprints and histology.

**FIG 1:**
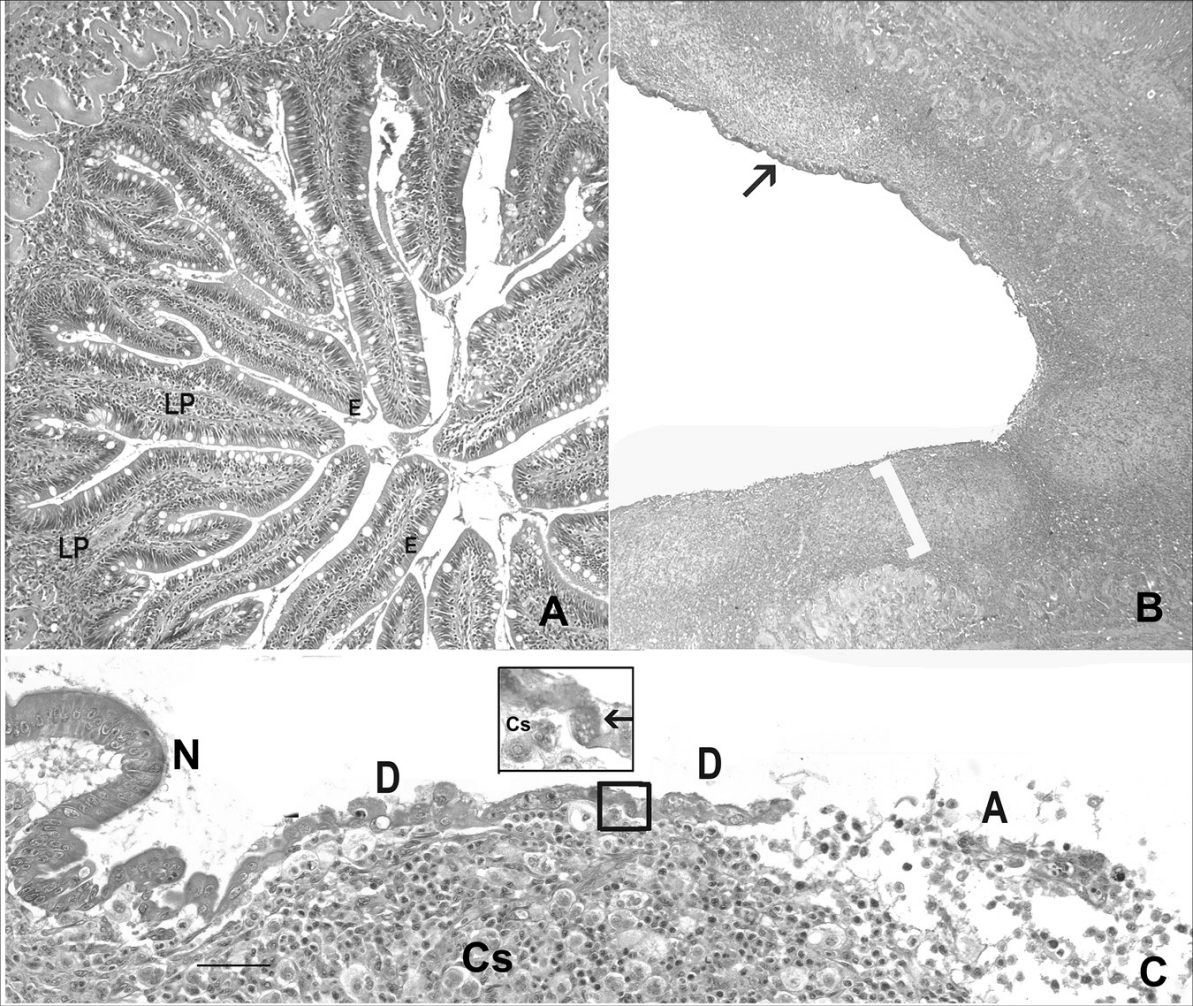
Pathologic changes in lower intestine in adult Chinook salmon. (A) Normal, with intact epithelium (E), prominent intestinal folds, and minimal expansion of lamina propria (LP). (B) Intestine with loss of intestinal folds, dyplasia, and flattening of the epithelium (arrow). The bracket indicates prominent expansion and inflammation of the lamina propria. (C) High magnification of lower intestine showing transition from normal epithelium (N), dysplasia (D), and complete loss of epithelium (A). Cs = Ceratonova shasta. The arrow in the inset indicates enterocytes infected with *Enterocytozoon schreckii* (hematoxylin- and eosin-stained). Bar = 20 μm.

Histological examination of the intestines of 30 adult Chinook salmon spawned at Willamette Hatchery in Oakridge, Oregon (43.9249°N, 122.8078°W) revealed prominent pathological changes in the lower intestine and pyloric ceca (Fig. 1). All of the fish exhibited profound expansion or inflammation of the lamina propria. The average percentage of epithelial loss was 44% (minimum, 15%; maximum, 98%) (Table 1). The remaining epithelium was often dysplastic, and sloughed enterocytes were observed in the lumen.

**TABLE 1:** Sex, coinfection, and intestinal epithelial loss in *E. schreckii*-infected and uninfected Chinook salmon examined in this study

	*Enterocytozoon schreckii* infection status	
	Infected	Uninfected
Male	6	9
Female	11	4
Infected with *Ceratonova shasta*	13	10
Epithelial loss (avg and 95% confidence interval) (%)	53 ± 12	60 ± 15

**FIG 2:**
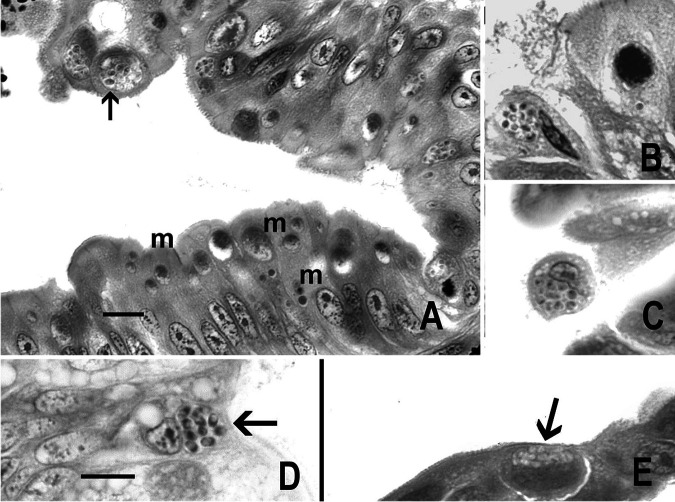
E. schreckii in intestinal epithelium of adult Chinook salmon. (A, B, C, E) Hematoxylin and eosin stained. (D) Giemsa stained. Bars = 10 μm. (A) Lower aspect of epithelium shows numerous presporogonic forms (meronts, m) toward distal aspect of relatively normal epithelium. The arrow indicates a possible interfacial membrane in sloughed enterocyte with developed spores. (B, C) Sloughed enterocytes with spores. (D) Developed spores (arrow) with posterior vacuoles. (E) Round epithelial cell with spores (arrow) in dysplastic epithelium.

High-magnification inspection of the epithelium, particularly dysplastic regions, revealed organisms suggestive of microsporidia in the genera Enterocytozoon or Enterospora in the distal cytoplasm of enterocytes of the intestinal epithelia of 17 of 30 (56%) fish examined by histology (Fig. 2). The microsporidia were observed as multinucleate meronts in the host cell cytoplasm (proliferative phase) and sporogonial plasmodia (sporogonal phase) containing sporoblasts, which ultimately develop into spores that are released from the host cell. The histology indicated the presence of an interfacial membrane surrounding the proliferative and sporogonal phases within the host cell. Spores were more frequently observed in dysplastic or sloughed enterocytes (Fig. 1 and 2), in contrast to regions of intact epithelium with normal, columnar enterocytes. Presporgonic forms appeared as basophilic spheres of various sizes and occurred within the distal aspects of enterocytes. Compared to spores, they were more common within normal, intact enterocytes. No significant difference in the prevalence of the parasite was detected between males and females (*t* test *P* = 0.27). There was no difference in the percentage of epithelium lost (*t* test *P* = 0.51) or the percentage of dysplastic epithelia (*t* test *P* = 0.90) between infected versus uninfected fish. A total of 23 of the 30 fish examined were also infected with by C. shasta, which proliferated in the lamina propria, and of these 15 had coinfections with the novel microsporidium.

**FIG 3:**
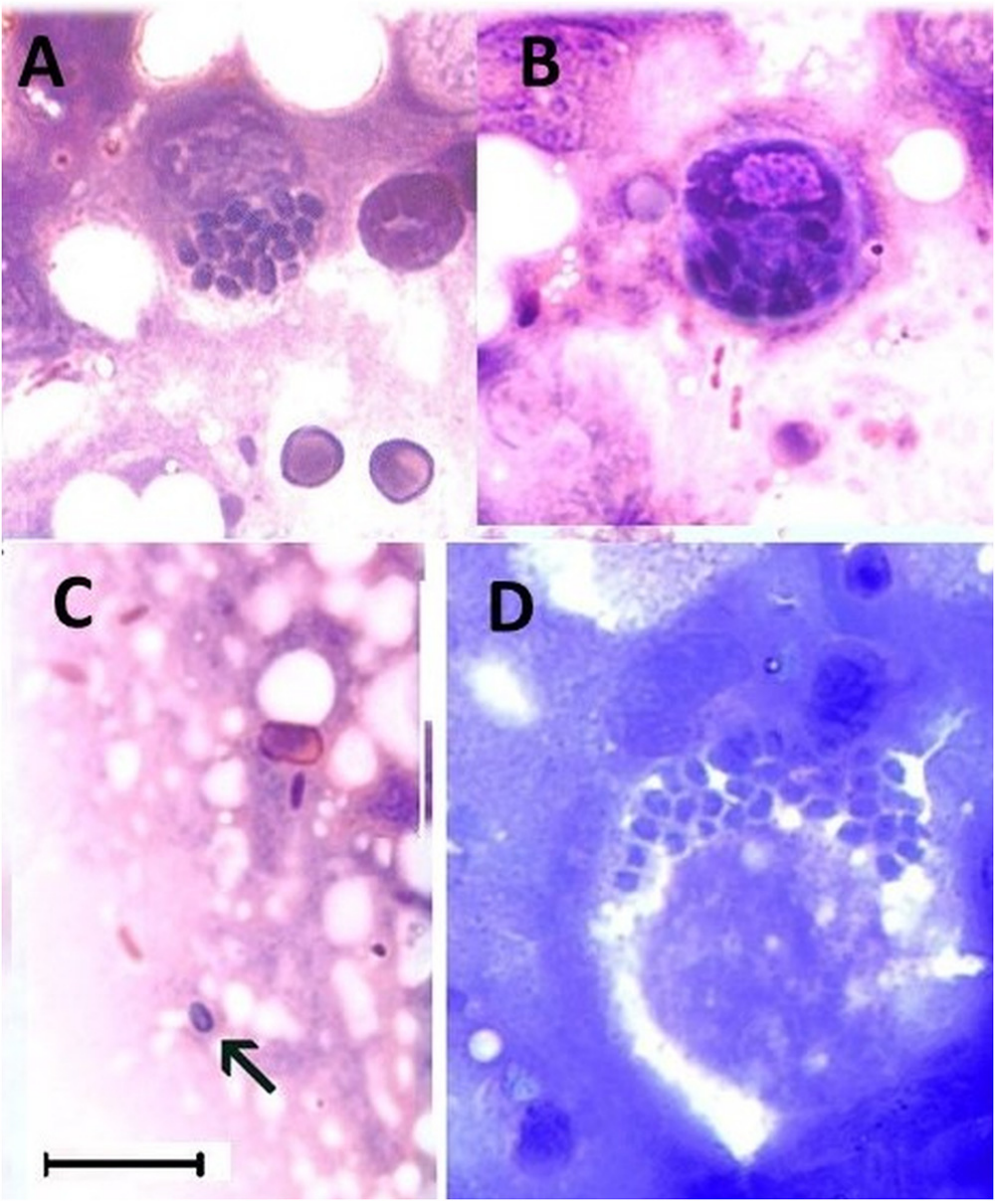
E. schreckii spores from imprints. (A to C) Gram stained. (D) Giemsa stained. (A, B, D) Aggregates of spores in presumptive enterocytes. (C) Gram-positive free spore (arrow). Bar = 10 μm.

**FIG 4:**
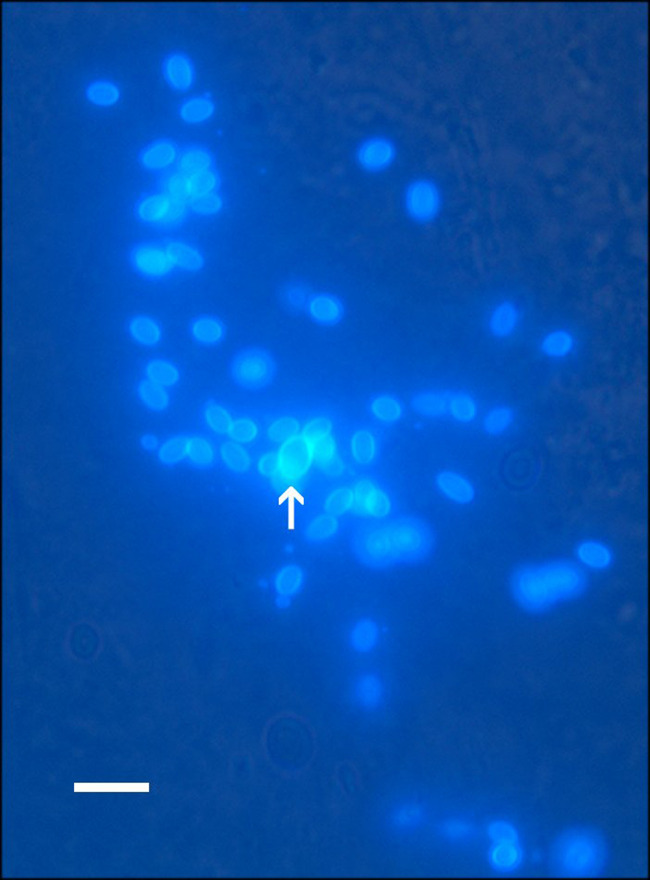
E. schreckii spores with fluorescence. Fungi-Fluor-stained spores visualized with 4′,6′-diamidino-2-phenylindole hydrochloride fluorescence. Bar = 10 μm; arrow = macrospore.

Touch prints detected microsporidia in a few fish (Fig. 3). Spores in touch prints appeared oval to subspherical and were Gram positive. Fungi-Fluor-stained imprints revealed spherical to oval spores that ranged in length from 2.0 to 2.5 μm (Fig. 4). A single macrospore identified among an aggregate of normal-sized spores measured 3.5 μm in length.

### Ultrastructure.

**FIG 5:**
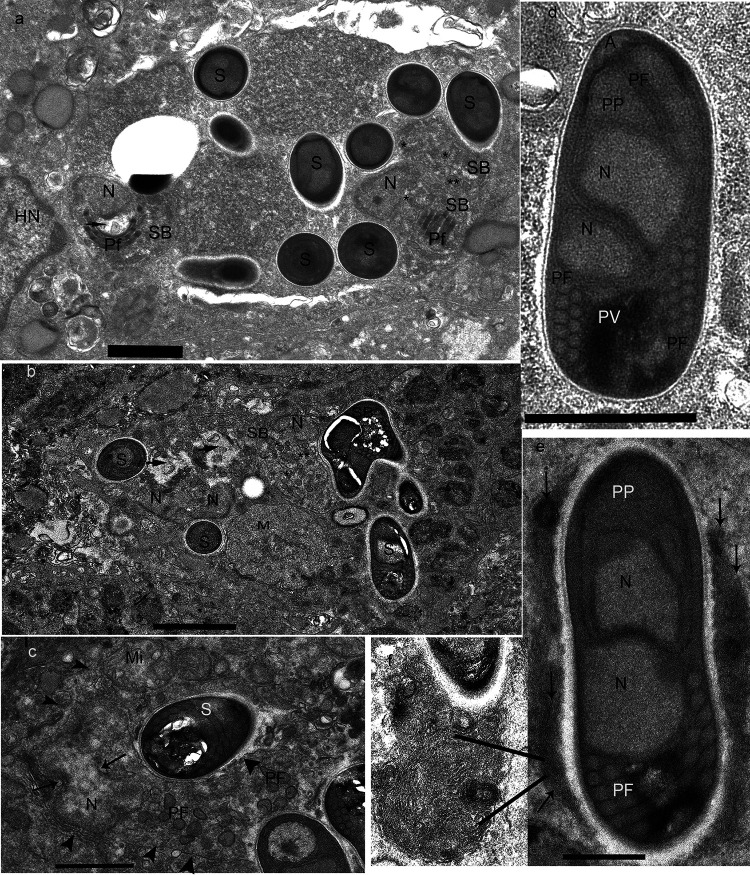
Transmission electron microscopic images of E. schreckii in the enterocytes of Chinook salmon. (a) Salmon enterocyte infected with E. schreckii. The host nucleus (HN) is in close proximity to a sporogonic plasmodium that is in direct contact with the host cytoplasm. Portions of the plasmodium appears to be composed of a dense matrix in which numerous electron-dense disks (EDD) (asterisks), which are precursors to polar filament formation (the polar tube is termed the polar filament when seen within the microsporidian cytoplasm), spores (S), and sporoblasts (SB) in different stages of development, are present. On the right side of the plasmodium are two sporoblasts (SB) in early development. The upper SB contains several tubular precursors (asterisk), the presence of which indicates the polar filament (Pf) is starting to form. In the adjacent SB, the tubular precursors (asterisk) are also present, and some EDDs appear to be combining, and several segments of PF have started to form. The SB near the HN has several partial PF coiled segments. The PF coils appear to be encircling an electron-lucent inclusion (ELI) (arrow) that is abutted to the SB nucleus. Several spores (S) are present in the plasmodium matrix indicating that the development of this organism is asynchronous. Bar = 1 μm. (b) A second plasmodium containing an early meront (M), several SBs with the PF just starting to develop, and spores (S). Additionally, a very-late-stage SB or early spore identified by the presence of a thin electron-lucent endospore coat and containing a coiled PF is present. Note the presence of EDDs (asterisk) and ELIs (arrows) next to the SB nucleus (N). Bar = 2 μm. (c) A third plasmodium containing an elongated sporont or early SB undergoing karyokinesis. Note the pinched-in nucleus (N) with spindle plaques (arrows). The elongated cytoplasm contains a few PF precursors. The parasite limiting membrane (arrowheads) is abutted by host mitochondria (Mi), and a mature spore (S) is also present. Bar = 1 μm. (d) Mature spore with a thin electron-lucent endospore coat. The spore contains an anchoring disc (A) attached to the straight or “manubrium” portion of the polar filament (PF) encompassed by a polaroplast (PP). In the middle of the spore are two abutted nuclei (N) in a typical diplokaryon arrangement. Note the well-defined nuclear membranes. The posterior of the spore contains a posterior vacuole (PV) and the coiled portion of the PF. The coil is arranged as a doublet, and the cross section cut through the PF coil reveals five and possibly six sets of doublets. Bar = 1 μm. (e) Sagittal section through a mature spore. The spore surface or exospore coat is covered with tightly packed membranes (arrows), with some appearing to be in clusters. This spore contains a polaroplast (PP) and nucleus (N) in a diplokaryotic arrangement, and five to six cross sections of the PF coil are visible. Bar = 0.5 μm. (f) High magnification of a portion of a membrane cluster attached to the spore. Note the tightly packed membranes arranged in an orderly manner. Some membranes appear to be forming circular or spherical clusters around an organizing site.

Transmission electron microscopic (TEM) examination of the enterocytes revealed the presence of several microsporidial spores and sporoblasts in various stages of development. Initially, the spores and sporoblasts appear to be located directly in the host cytoplasm. Closer examination revealed that the parasites were in a dense matrix within a sporogonial plasmodium, whose outermost surface is difficult to discern because it is in direct contact with the host cell cytoplasm. Some of the plasmodial outer surfaces appeared to separate from the host cytoplasm as a result of a fixation or dehydration artifact, enabling identification of the parasite-host interface (Fig. 5a). Neither pansporoblastic membranes or a parasitophorous vacuole was observed on any of the plasmodia. The sporoblasts, depending on their stage of development and orientation inside the thin section, appeared to have a single nucleus, electron-lucent and -dense oval structures, precursor polar filament segments, and formed polar filaments (the polar tube is termed the polar filament when seen within the microsporidian cytoplasm). This formation of the polar filament and other parts of the microsporidial extrusion apparatus before division into individual sporoblasts is atypical of most microsporidia but suggests that this organism’s development is consistent with the Enterocytozoonidae (Fig. 5a). In most plasmodia, early meronts, early- and late-stage sporoblasts, and mature spores are present, indicating asynchronous development. On occasion, early sporoblasts appear to be undergoing karyokinesis as evidenced by the presence of a pinched in nucleus and nuclear spindle plaques (Fig. 5c). This nuclear division suggests disporoblastic development or the formation of diplokaryon nuclear arrangement.

The mature spores (Fig. 5a and b) found both in the sporogonial plasmodia and free in the host cytoplasm appear to have an electron-dense exospore wall overlying an unusually thin electron-lucent endospore coat. The anterior tip of the spore contains a mushroom-shaped anchoring disc connected to a relatively straight portion of polar filament, which is encircled by the polaroplast. Distal to the polaroplast are two nuclei in a typical diplokaryotic arrangement indicated by the presence of double membranes between the nuclei. The polar filament coiling appears to start at the level of the diplokaryon and continues its coiling to the posterior end of the spore. Cross sections of the filament coil reveal that it is arranged as a doublet within the spore, and five or possibly six sets of staggered doublet coils are visible in section. The spores also contain a well-formed posterior vacuole encircled by some of the filament coils (Fig. 5d). Some spores were present in a mass of tightly packed electron-dense material within the host cell cytoplasm. This material appeared to be membranous and attached to the exospore wall (Fig. 5e). Higher magnification revealed that the membranes formed circular clusters around organizing sites (Fig. 5f).

**FIG 6:**
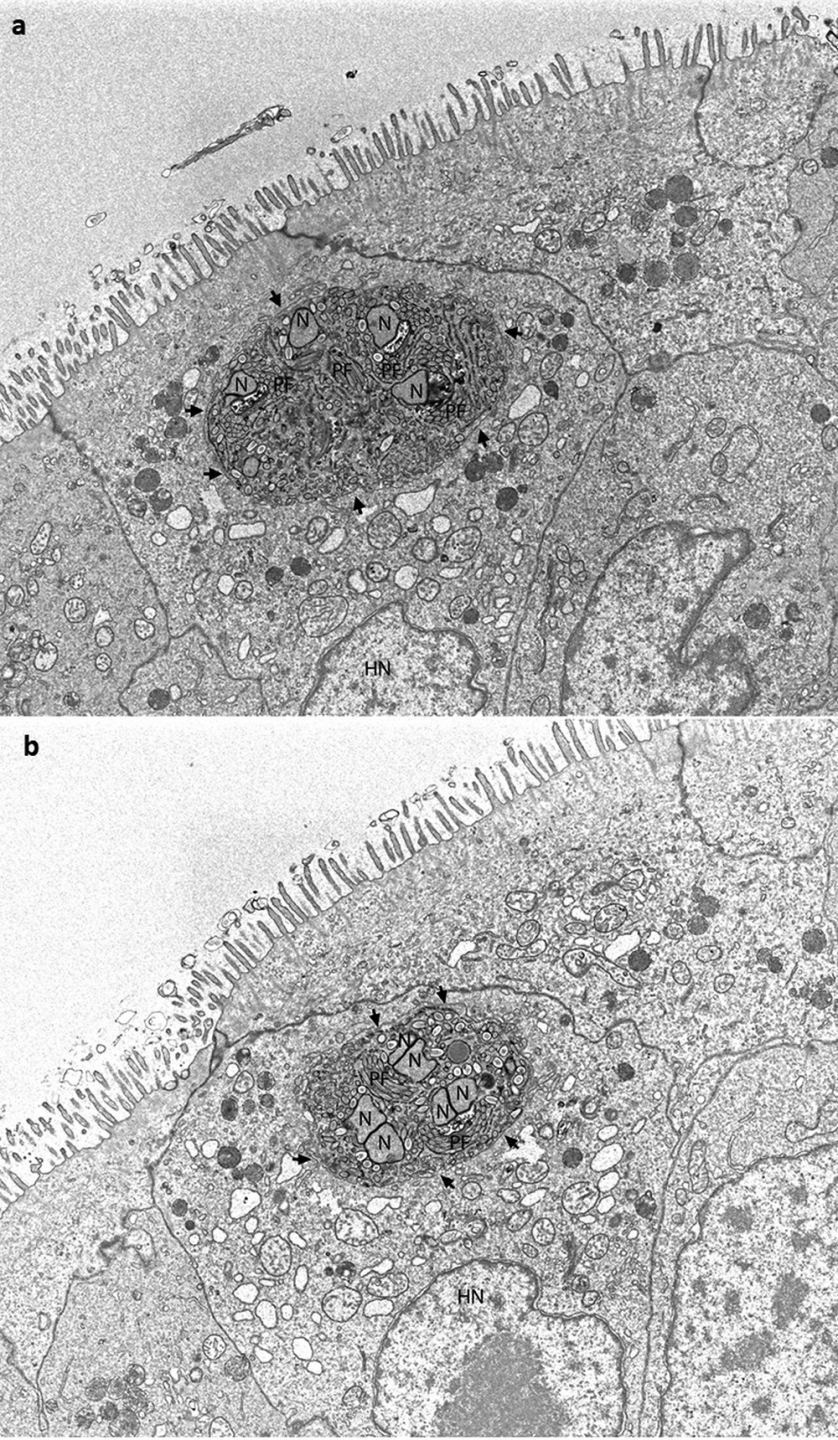
Array tomography images of E. schreckii in the enterocytes of Chinook salmon. (a) Image of ultrathin (55 nm) section 11 of 40 of an E. schreckii-infected enterocyte. In this section, the sporogonial plasmodium contains four uninucleate sporoblasts (SB), each surrounded by electron-dense and -lucent discs or sacs that are precursors of the microsporidial extrusion apparatus. Several rows of longitudinal and cross sections of the polar filament (PF) coil (the polar tube is termed the polar filament when seen within the microsporidian cytoplasm) are visible adjacent to the sporoblast nucleus (N), indicating that the developing SBs have various orientations within the plasmodium. The plasmodium appears to be bounded by a very thin membrane in direct contact with the host cell cytoplasm (arrowheads). Note the presence of microvilli on the enterocyte surface and orientation of this cell. Bar = 1 μm. (b) Image of ultrathin (55 nm) section 31 of 40, of an *E. schreckii*-infected enterocyte. In this section, the sporogonial plasmodium has three visible sporoblasts (SB), each containing two nuclei (N) in a diplokariotic arrangement. Several segments of the polar filament (PF) coil are visible and appear to be organized into rows opposite the diplokaryon. This portion of the plasmodium continues to be in direct contact with the host cytoplasm (arrowheads), confirming that this parasite does not develop in pansporoblast or a parasitophorous vacuole. Note that this section is approximately 1,100 nm deeper into the cell than slice 11.

To elucidate the structure of developing organisms and their organization inside the plasmodium, array tomography (51) was utilized. A total of 40 ultrathin (55 nm) serial sections of enterocytes were collected and then imaged with a scanning electron microscope (SEM) in backscatter mode. The 40 images were then electronically stacked, enabling visualization of a total *Z* depth of 2.2 μm. Fig. 6 is an image of section 11 from the stack and contained an infected enterocyte that could be identified by its well-defined microvilli, basal located nucleus, and adjoining cells with tight junctions. The enterocyte contained a sporogonial plasmodium in direct contact with the host cytoplasm. The plasmodium contained developing microsporidial structures that included sporoblasts, electron-dense disks (EDDs), and electron-lucent disks (ELDs) or sacs that are precursors of the extrusion apparatus. Several stacks of longitudinal and cross sections of the polar filament coil adjacent to the sporoblast nucleus were evident. The presence of both long and cross sections of polar filament coils indicates that the developing stages are oriented in multiple positions within the plasmodium. The sporoblast nucleation appeared to be uninucleate, suggesting that the mature spores would be uninucleate (Fig. 6a). This image was in direct opposition to the TEM-imaged spores (Fig. 5d and e) that contained diplokaryotic nuclear arrangements. Initially the presence of uninucleate sporoblasts from the array tomography samples suggested that there might be two different spore types or possibly two different infections/species. Because array tomography enables one to examine individual sections from different locations/depths within the three-dimensional stack, we were able to visualize section 31, which was 1.1 μm deeper into the plasmodium than section 11. Section 31 (Fig. 6b) contained three sporoblasts; each one had two nuclei (N) in a diplokariotic arrangement. Two of the sporoblasts had several segments of the polar filament coil organized into rows opposite the diplokaryon. The third sporoblast was surrounded by both electron-lucent or empty looking-vesicles, and dense saccules that are precursors of early PF formation. The outer edge of the plasmodium continues to be in direct contact with the host cytoplasm, confirming that this parasite does not develop in pansporoblast or a parasitophorous vacuole.

Based on ultrastructural images obtained from both the TEM and array tomography of this microsporidial infection in the enterocytes of Chinook salmon, we demonstrate that this parasite does not develop a pansporoblast, nor does it develop in a parasitophorous vacuole but resides in direct contact inside its host cell cytoplasm. The plasmodium contains electron-lucent and -dense inclusions, vesicles, and discs associated with precocious polar filament development, the presence of five or six sets of polar tube doublets within spores and sporoblasts in cross section cuts, and the presence of diplokariotic nuclear arrangement in both the sporoblasts and spores, indicating that this parasite has all the morphological and developmental characteristics associated with the Enterocytozoonidae.

### Molecular phylogeny.

**FIG 7:**
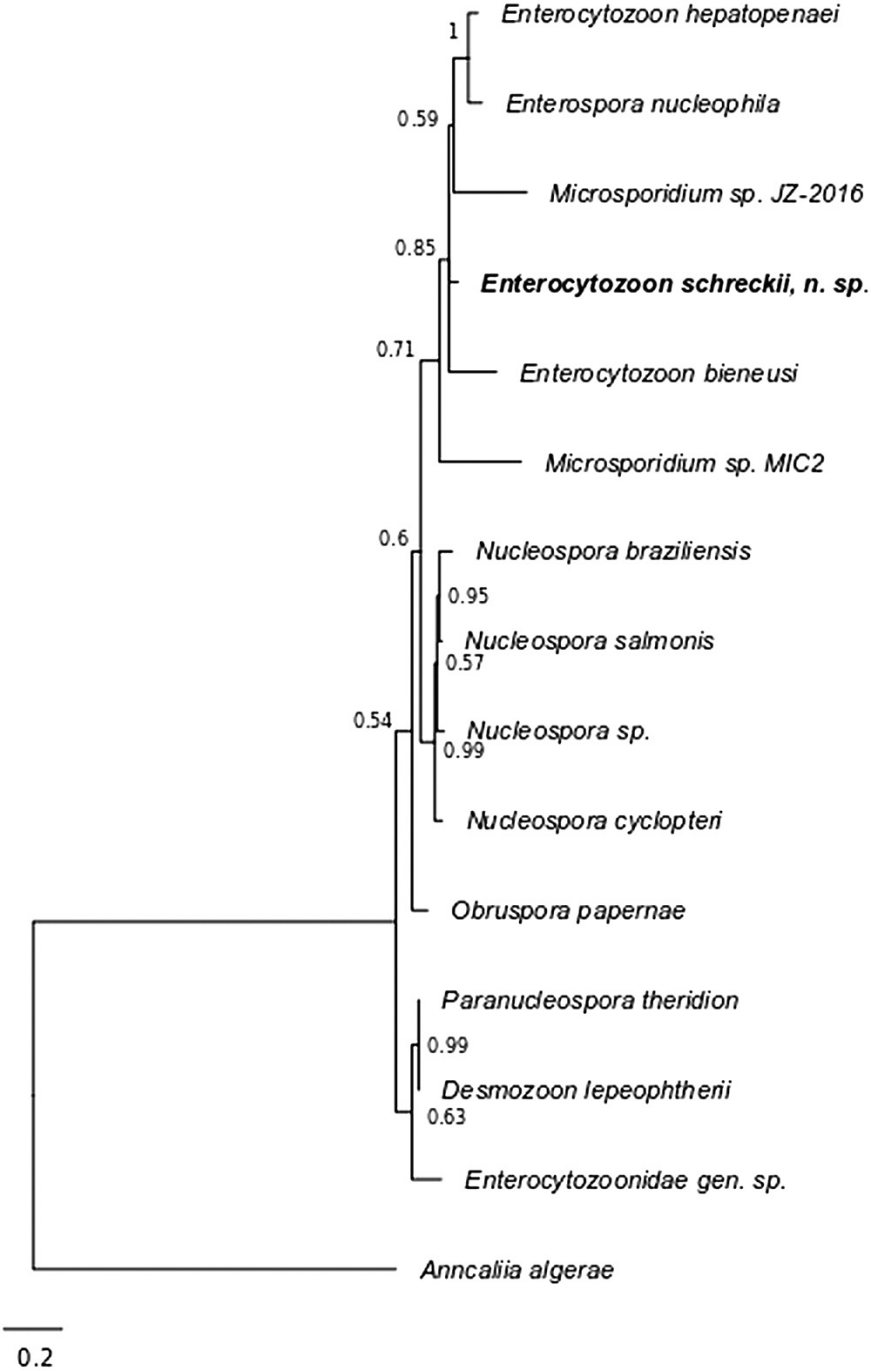
Phylogenetic relationships of E. schreckii and selected members of the Enterocytozoonidae. Phylogeny is based on small-subunit rRNA gene sequences and was generated using MrBayes version 3.2 using a GTR substitution model with gamma-distributed rate variation across sites and a proportion of invariable sites. Branch length is drawn to scale. Nodes are labeled with bootstrap values.

Using *Enterocytozoon*-specific primers (26), an ∼1,000-bp DNA fragment was amplified from intestinal epithelial scrapings of three fish. The products of two different PCRs from the same individual were sequenced entirely, resulting in a quality-trimmed 977-bp consensus sequence. The first two species identified in the top BLAST hits were Enterocytozoon hepatopenaei (91.4% identity) and Enterospora nucleophila (89.6% identity) based on small-subunit rRNA sequences. The Chinook salmon microsporidium resolved within the *Enterocytozoon* group Microsporidia (EGM) as defined by Stentiford et al. (27) and shared a most recent common ancestor with the subclade containing E. hepatopenaei and E. nucleophila and the subclade containing E. bieneusi (87% identity) and E. canceri (87.9% identity), as well as with Microsporidium JZ-2016 (83.4% identity), a novel microsporidia species isolated from farmed groupers (26) (Fig. 7).

### Taxonomic summary.

Phylum: Microsporidia (Balbiani, 1882).

Class: Terresporidia (Vossbrink and Debrunner-Vossbrinck, 2005).

Order: Microsporida (Balbiani, 1882).

Family: Enterocytozoonidae (Cali and Owen, 1990).

Genus: Enterocytozoon (Desportes, Le Charpentier, Galian, Bernard, Cochand-Priollet, Lavergne, Ravisse, and Modigliani, 1985).

Species: Enterocytozoon schreckii n. sp.

Diagnosis of the species: Histology **–** Confined to epithelium, earliest forms appear as very small punctate, basophilic homogenous spheres that enlarge as they mature. Later forms, presumably sporogonic forms, appear as lighter stained spheres containing structures consistent with developing spores. Spores are subspherical (Fig. 2B and C), stain positive with Giemsa, and contain a small posterior vacuole (Fig. 2D). Imprints **–** Gram positive with Gram stain. Fungi-Fluor-stained spores are subspherical, range from 2 to 2.5 μm in length and 1.5 to 2 μm in width (n = 40), and occur in aggregates of 15 to 30 spores within cytoplasm. Electron microscopy **–** Sporogenesis occurs within sporogonial plasmodia with no detectable interfacial membrane. (Fig. 5). During development, polar tube precursors are formed prior to plasmodial division into spores. Spores are ovoid and diplokaryotic. Fixed spores are 1.7 × 1.1 um in size, with the filament arranged in five or six sets of staggered doublet coils.

Type host: O. tshawytscha (Teleostei: Salmoniformes).

Site of infection: intestinal epithelium.

Prevalence: 17 of 30 (56%) in type locality collected in September 2020.

Type locality: Willamette Hatchery, near Oak Ridge, Oregon, United States (43.9249°N, 122.8078°W).

Etymology: The species epithet schreckii is given in reference to the internationally recognized fisheries scientist, Carl B. Schreck.

Type material deposited: Syntypes – Histologic slides stained with hematoxylin and eosin (U.S. National Parasite Collection). Gene sequence – Sequences comprising 977 bp of the small-subunit rRNA gene of E. schreckii have been deposited in the GenBank database under accession number OL780325.

Amended diagnosis of genus: Enterocytozoon (Deportes, Charpentier, Galian, Bernard, Cochhand-Priollet, Lavergne, Ravisse, and Modigliani, 1985): The microsporidia included in the genus Enterocytozoon are unified by a small-subunit rRNA gene-based molecular phylogeny and developmental characteristics. At least some development occurs in direct contact with the host cytoplasm. An interfacial membrane may or may not be present. Proliferative cells develop into sporogonial plasmodia, which produce sporoblasts by multiple fission. The extrusion apparatus is formed before fission of the sporogonial plasmodia. Spores lack a well-developed endospore and contain an electron-lucent vacuole. This genus occurs in a wide range of aquatic and terrestrial hosts, including crustaceans, fish, and mammals. The genus Enterocytozoon is phylogenetically distinct from *Nucleospora*, *Paranucleospora*, and *Desmozoon* based on small-subunit rRNA gene and is developmentally distinguished from *Nucleospora* in which development is restricted to the nucleus. Enterospora is a junior synonym of Enterocytozoon.

## DISCUSSION

The discovery of E. schreckii n. sp. contributes to clarifying the phylogenetic and taxonomic relationships within family Enterocytozoonidae and highlights the paraphyletic nature of genus Enterospora. Moreover, we observed morphological similarities between E. schreckii and members of genera Enterocytozoon and Enterospora (Palenzuela et al. [28] provide a review of the morphologic and host characteristics of these genera). TEM examination of numerous sections from multiple samples of Chinook salmon enterocytes observed in the current study demonstrate the presence of microsporidian developmental stages and mature spores in the enterocyte cytoplasm. Despite extensive examination of enterocyte nuclei in cells with and without cytoplasmic infections, no nuclear infections were observed. This cytoplasmic development is characteristic of the Enterocytozoon, as are the precocious polar filament formation from electron-dense discs, the presence of sporoblasts before division of the sporogonial plasmodium, and the polar filament arrangement observed in E. schreckii. However, although the developing stages appear to be uninucleate, examination with array tomography revealed that the sporoblast nuclear configuration is two nuclei tightly abutted by intact individual nuclear membranes, and this diplokaryotic arrangement resembles that of Enterospora nucleophilia rather than the monokaryotic arrangements observed in either of the previously described Enterocytozoon species.

The phylogenetic placement of E. schreckii further highlights the paraphyletic nature of the clade containing Enterocytozoon and Enterospora. The type species of Enterocytozoon is E. bieneusi, which was first described in AIDS patients in 1985 (6) and has since been identified in several mammal and bird species. A second member of the genus, E. hepatopenaei, was later identified in the hepatopancreatic tubules of black tiger shrimp Penaeus monodon (29). Members of Enterocytozoon develop in direct contact with the host cell cytoplasm but closely abut the nucleus. In contrast, members of Enterospora display at least some intranuclear development but are grouped phylogenetically with Enterocytozoon rather than with other taxa exhibiting intranuclear development (i.e., Nucleospora, Paranucleospora, and Desmozoon). The type species of Enterospora, E. canceri, was first identified in the European edible crab (Cancer pagurus) (30). Like E. hepatopenaei, E. canceri infects the hepatopancreatic tubules of a marine crustacean but is distinguished by its purely intranuclear development. A second species of Enterospora, E. nucleophila, infects the enterocytes and rodlet cells at the intestinal epithelium of gilthead sea bream (Sparus aurata), a commercially important aquaculture species (28). Unlike E. canceri, E. nucleophila exhibits some cytoplasmic development in addition to intranuclear development. However, E. nucleophila appears more closely related to E. hepatopenaei than to E. canceri based on SSU rRNA gene sequence comparisons.

The designation of Enterospora as a separate genus is called into question by the divergent ecology and phylogeny of the two described species within this genus. E. schreckii further highlights the questionable taxonomic divisions within this clade, because it shares a most recent common ancestor with members of both Enterocytozoon and Enterospora and shares biological and ecological features with E. nucleophila, as they both infect the intestinal epithelia of teleost fishes and share similar morphology (e.g., diplokaryotic nuclear arrangement). Overall, our results support the redescription of Enterocytozoon to include Enterospora as a junior synonym due to rules of nomenclature priority.

Enterocytozoon and related microsporidia (Enterocytozoon group Microsporidia, EGM) frequently infect the gastrointestinal tracts of immunocompromised fish, birds, mammals, and invertebrates via food- and water-born transmission and cell-cell autoinfection (27). E. schreckii fits within this paradigm as it occurs in the gastrointestinal tracts of Chinook salmon during migration and sexual maturation, a life history stage that is characterized by intense energetic demands (31), elevated stress hormones (32), and diminished immune responses (21). The exact mechanisms by which the EGM exploit host immune dysfunction are unknown, but gut mucosal barriers are known to be disrupted by multiple internal and external forces, including physical or psychological stressors, aging, and immunopathology, all of which could provide gateways to opportunistic EGM infection (27). Some species, including E. bieneusi, are thought to persist asymptomatically in immunocompetent hosts at undetectable levels and then emerge as active infections when host immunity is compromised (33). This may be the case with *E. schrekii* as adult Chinook salmon experience elevated stress hormones and altered immune capacity during migration in freshwater. Latent infections that were acquired in earlier developmental stages may not become symptomatic until the highly stressful return migration and concurrent physiological changes. The life stage at which Chinook salmon become infected with E. schreckii is unknown, but another EGM species, Nucleospora salmonis, is known to infect salmonids during the freshwater juvenile stages and marine stages of anadromous salmonids. It has been proposed that members of EGM act as sentinels of host immune competence across diverse environments and host taxa (2, 27). Under this framework, the emergence of E. schreckii is notable as it coincides with increasingly stressful environmental conditions imposed on migrating Chinook salmon by climate change (34), infrastructure (35), aquaculture (36), and other anthropogenic stressors.

An intriguing parallel between E. bieneusi and E. schreckii is that both are prominent in hosts undergoing immunosenescence. Across vertebrate taxa, aging, and senescence associate with elevated cortisol (37, 38), gut mucosa damage (39), and loss of immunoregulatory capacity (40, 41). These traits are also evident both in human AIDS patients (20, 42) and in sexually mature Chinook salmon (38) and may therefore contribute to Enterocytozoon susceptibility, particularly loss of gut mucosal barrier integrity (27). A histopathologic hallmark of AIDS and other immunosuppressive disorders (e.g., common variable immunodeficiency [43]) is the loss of villous structure and atrophy of the epithelium, with concurrent severe inflammation in the underlying mucosa. These features were also observed in the intestines of sexually mature Chinook salmon in the current study. In addition, Enterocytozoon infection may lead to further immunosuppression and facilitate coinfection by other parasites and pathogens (44). Chinook salmon often experience numerous coinfections during migration and sexual maturation (15, 16); therefore the ecological role of E. schreckii in salmonid parasite communities warrants further study.

Variation in the timing of senescence is of critical importance to Chinook salmon populations because accelerated senescence that leads to death prior to spawning results in zero lifetime fitness for individuals. At the population level, prespawning mortality correlates with environmental stressors such as elevated water temperature and the presence of hatchery-origin fish (36). However, identifying individual-level predictors of prespawning mortality has proved elusive. Based on the distinct biological parallels between E. bieneusi and E. schreckii and the known tendency of EGM and other microsporidia to infect immunocompromised hosts, it is possible that E. schreckii presence or abundance could serve as a biomarker for immunosenescence and hence prespawning mortality risk in Chinook salmon. Currently, it is unknown whether E. schreckii associates directly with senescence *per se*, but given that it has only been observed in migrating adults as they approach sexual maturation and spawning, infection status may associate with individual variation in prespawning mortality risk.

From a One Health perspective, elucidating the biology, pathology, and phylogenetics of E. schreckii contributes to our understanding of the EGM and other microsporidia as sentinels of environmental stressors and population health in a changing world (45). Because our findings suggest that E. schreckii infection in Chinook salmon mirrors that of E. bieneusi in human AIDS patients, further study of E. schreckii may lead to a deeper understanding of the ecology and biology of both host-parasite systems, thus contributing to both human and animal health research. Future study of E. schreckii will focus on clarifying the relationship between infection, senescence, and mortality.

## MATERIALS AND METHODS

### Sample collection.

Due to the wide range of infectious agents that are prevalent in returning Chinook salmon and variation in the location and severity of infection (15), postmortem histopathological examination is often used to assess infection status in the carcasses of PSM and postspawned fish. During histopathological examination of Chinook salmon carcasses collected at Willamette Hatchery on a tributary of the Willamette River in Oakridge, Oregon (43.9249°N, 122.8078°W), a small, microsporidian-like parasite was identified in the intestinal epithelium. After initial identification of the parasite, we collected intestinal tissues of 30 fish carcasses for histology and light microscopy. These fish were originally collected in July 2020 at Dexter Dam near Dexter, Oregon (43.9129°N, 122.7880°W) by the Oregon Department of Fish and Wildlife (ODFW) and kept at the hatchery in holding ponds with nonrecirculating river water flowthrough until September 2020 when they were euthanized and artificially spawned by ODFW.

### Histology and light microscopy.

For histological analysis, intestines were cut into transverse cross sections, and representative pieces were preserved in Dietrich’s fixative, embedded in paraffin, sectioned, and stained with hematoxylin and eosin or Giemsa by the Oregon Veterinary Diagnostic Laboratory (Corvallis, OR). To visualize whole spores, touch preparations were prepared from small (approximately 10 mm) sections of fresh intestine that were cut lengthwise to expose the internal gut epithelium. Touch preparations were stained with either Giemsa or Gram stains. For fluorescence staining, frozen intestinal samples were thawed and cut lengthwise. The epithelial layer was scraped and smeared onto a glass slide and then stained with Fungi-Fluor (Polysciences Inc., Warrington, PA, catalog no. 17442-1), counterstained with solution B (blocking agent) for 1 min, and rinsed with distilled water. The slides were observed as water mounts under sealed coverslips using a fluorescence microscope with a 4′,6′-diamidino-2-phenylindole hydrochloride (DAPI) filter (250 to 400 nm).

### Electron microscopy.

Intestinal enterocytes were obtained by scraping gut epithelial cells from the fish intestines. For transmission electron microscopy (TEM), the cell scrapings were fixed in 2.5% glutaraldehyde in 0.1 M sodium cacodylate buffer and stored overnight. The samples were rinsed in 0.1 M sodium cacodylate buffer and postfixed in 1% buffered OsO_4_ overnight. The postfixed samples were dehydrated through a graded series of ethanols, transitioned into propylene oxide, and embedded in Epon LX-112 resin (LADD Research Industries, Burlington VT). Thin sections (70 to 80 nm) were cut on a Leica UC7 ultramicrotome (Leica, Morrisville, NC) and then stained with uranyl acetate and lead citrate. The samples were observed with an FEI Tecnai 12 TEM (FEI, Hillsboro, OR) operated at 80 KV, and the images were recorded with a OneView 16 Megapixel digital camera (Gatan, Pleasanton, CA) at the Rutgers Electron Microscopy Facility (Newark, NJ).

For array tomography (46), intestinal enterocyte specimens were processed by the methods of Deerinck et al. (47). Briefly, the samples were fixed with 2% paraformaldehyde and 2.5% glutaraldehyde in 0.1 M sodium cacodylate buffer, postfixed with freshly prepared 2% osmium tetroxide, 1.5% potassium ferrocyanide, 0.15 M sodium cacodylate, 2 mM CaCl_2,_ followed by 1% thiocarbohydrazide, and then 2% osmiun tetroxide and then bloc stained with 1% uranyl acetate and further stained with lead aspartate. The samples were then dehydrated through a graded series of ethanols and embedded in LX112 resin (LADD Research Industries, Burlington, VT). Ultrathin (55 nm) sections were cut on a Leica ARTOS 3D ultramicrotome and collected onto glass coverslips. The sections were examined in a Zeiss Supra 40 field emission scanning electron microscope (Carl Zeiss Microscopy, LLC), in backscatter mode using an accelerating voltage of 8.0 KV. Regions of interest were collected using ATLAS 5.0, with a pixel size of 6.0 and a dwell time of 6 us. Stacks were aligned using IMOD. This aspect of the study was performed at the Albert Einstein Analytical Imaging Facility (Bronx, NY).

### Phylogenetic analysis.

Frozen intestinal tissue samples from several fish that were positive for the parasite by histology were thawed, and epithelial cells were scraped from the intestinal wall for analysis. DNA was extracted from scraped material using the DNEasy blood and tissue kit (Qiagen, Valencia, CA, catalog no. 69504) and the manufacturer’s protocol. We used the Enterocytozoonidae-specific primers designed by Xu et al. (26) to target an ∼1,000-bp segment of the small-subunit rRNA gene. PCR was carried out in a 50-μl reaction volume using 2 μl of template, 5 μl of PCR buffer, 1.5 μl MgCl_2_, 1 μl of 10 mM dNTP mix, 1 μl each of 10 μM primer, and 0.5 μl of polymerase. Cycling conditions consisted of an initial denaturation (3 min at 95°C) and 35 cycles (94°C for 40 s, 59°C for 30 s, and 72°C for 90 s) followed by a final extension of 10 min at 72°C. We successfully amplified ∼1,000-bp products from three of the seven extracted samples, and the PCR products were purified using QIAquick PCR purification kits (Qiagen, Valencia, CA, catalog no. 28104). Sanger sequencing was performed on each isolate with forward and reverse primers using a 3730 capillary sequence machine (Thermo Fisher Scientific, Waltham, MA, catalog no. 4331250).

The resultant reads were assembled and trimmed in MegaX (48) and aligned with SSU sequences from representative members of family Enterocytozoonidae using MUSCLE (49) (Table 2). A phylogenetic tree was generated in MrBayes version 3.2 (50) using a GTR substitution model with gamma-distributed rate variation across sites and a proportion of invariable sites. The Markov chain Monte Carlo simulation was run for 1,000,000 generations resulting in a standard deviation of split frequencies below 0.001.

**TABLE 2:** Microsporidia isolates included in the phylogenetic analysis of the *Enterocytozoon schreckii* SSU rRNA gene.

Species	Host	GenBank accession no.
Desmozoon lepeophtherii	Salmon louse and Atlantic salmon	AJ431366.2
Enterocytozoon bieneusi	Humans and other terrestrial vertebrates	L16868.1
Enterocytozoon hepatopenaei	Black tiger shrimp	KY593127.1
Enterocytozoonidae gen. sp.	Sole	AF201911.1
Enterospora nucleophila	Gilt-head bream	KF135645.1
Microsporidium sp. JZ-2016	Grouper	KR263870.1
Microsporidium sp. MIC2	Daphnia	FJ794873.1
Nucleospora braziliensis	Nile tilapia	MW491352.1
Nucleospora cyclopteri	Lumpfish	KC203457.1
Nucleospora salmonis	Salmonids	U78176.1
Nucleospora sp.	Eel	JN938583.1
Obruspora papernae	Blotchfin dragonet	HG005137.1
Paranucleospora theridion	Atlantic salmon	FJ594981.1
Enterospora canceri	European edible crab	HE584634.1
Anncaliia algerae	Arthropods and humans (outgroup)	AF069063.1
